# Model-based cardiovascular monitoring of acute pulmonary embolism in porcine trials

**DOI:** 10.1186/cc9437

**Published:** 2011-03-11

**Authors:** JA Revie, DJ Stevenson, JG Chase, CE Hann, BC Lambermont, A Ghuysen, P Kolh, GM Shaw, T Desaive

**Affiliations:** 1 University of Canterbury, Christchurch, New Zealand; 2 University of Liege, Belgium; 3 Christchurch Hospital, Christchurch, New Zealand

## Introduction

Diagnosis and treatment of cardiac and circulatory dysfunction can be error-prone and relies heavily on clinical intuition and experience. Model-based approaches utilising measurements available in the ICU can provide a clearer physiological picture of a patient's cardiovascular status to assist medical staff with diagnosis and therapy decisions. This research tests a subject-specific cardiovascular system (CVS) modelling technique on measurements from a porcine model of acute pulmonary embolism (APE).

## Methods

Measurements were recorded in five pig trials, where autologous blood clots were inserted every 2 hours into the jugular vein to simulate pulmonary emboli. Of these measurements only a minimal set of clinically available or inferable data were used in the identification process (aortic and pulmonary artery pressure, stroke volume, heart rate, global end diastolic volume, and mitral and tricuspid valve closure times). The CVS model was fitted to 46 sets of data taken at 30-minute intervals (*t *= 0, 30, 60, ..., 270) during the induction of APE to identify physiological model parameters and their change over time in APE. Model parameters and outputs were compared with experimentally derived metrics and measurements not used in the identification method to validate the accuracy of the model and assess its diagnostic capability.

## Results

Modelled mean ventricular volumes and maximum ventricular pressures matched measured values with median absolute errors of 4.3% and 4.4%, which are less than experimental measurement noise (~10%). An increase in pulmonary vascular resistance, the main hemodynamic consequence of APE, was identified in all the pigs and related well to experimental values (*R *= 0.68). Detrimental changes in reflex responses, such as decreased right ventricular contractility, were noticed in two pigs that died during the trial, diagnosing the loss of autonomous control. Increases in the ratio of the modelled right to left ventricular end diastolic volumes, signifying the leftward shift of the intraventricular septum seen in APE, compared well with the clinically measured index (*R *= 0.88).

## Conclusions

Subject-specific CVS models can accurately and continuously diagnose and track acute disease-dependent cardiovascular changes resulting from APE using readily available measurements. Human trials are underway to clinically validate these animal trial results.

